# SIRT1 protects against myocardial ischemia–reperfusion injury via activating eNOS in diabetic rats

**DOI:** 10.1186/s12933-015-0299-8

**Published:** 2015-10-21

**Authors:** Mingge Ding, Jingyi Lei, Hongcheng Han, Weibo Li, Yinxian Qu, Enqing Fu, Feng Fu, Xiaoming Wang

**Affiliations:** Department of Geriatrics, Xi’an Central Hospital, Xi’an, 710000 China; Department of Cardiology, Xi’an Central Hospital, Xi’an, 710000 China; Department of Geriatrics, Xijing Hospital, Fourth Military Medical University, 15 Changlexi Road, Xi’an, 710032 China; Department of Respiratory Medicine, Tangdu Hospital, Fourth Military Medical University, Xi’an, 710032 China; Department of Physiology, Fourth Military Medical University, 169 Changlexi Road, Xi’an, 710032 China

**Keywords:** SIRT1, Ischemia–reperfusion, Diabetes, Oxidative stress, eNOS

## Abstract

**Background:**

Diabetic patients are more sensitive to myocardial ischemic injury than non-diabetic patients. Silent information regulator 1 (SIRT1) is a nicotinamide adenine dinucleotide-dependent histone deacetylase making the heart more resistant to ischemic injury. As SIRT1 expression is considered to be reduced in diabetic heart, we therefore hypothesized that up-regulation of SIRT1 in the diabetic heart may overcome its increased susceptibility to ischemic injury.

**Methods:**

Male Sprague–Dawley rats were fed with high-fat diet and injected with streptozotocin once to induce diabetes. Diabetic rats received injections of adenoviral vectors encoding SIRT1 (Ad-SIRT1) at five myocardial sites. Four days after adenoviral injection, the rats were subjected to myocardial ischemia and reperfusion (MI/R). Outcome measures included left ventricular function, infarct size, cellular death and oxidative stress.

**Results:**

Delivery of Ad-SIRT1 into the hearts of diabetic rats markedly increased SIRT1 expression. Up-regulation of SIRT1 in diabetic hearts improved cardiac function and reduced infarct size to the extent as in non-diabetic animals following MI/R, which was associated with reduced serum creatine kinase-MB, lactate dehydrogenase activities and cardiomyocyte apoptosis. Moreover, Ad-SIRT1 reduced the increase in the superoxide generation and malonaldialdehyde content and simultaneously increased the antioxidant capability. Furthermore, Ad-SIRT1 increased eNOS phosphorylation and reduced eNOS acetylation in diabetic hearts. NOS inhibitor L-NAME inhibited SIRT1-enhanced eNOS phosphorylation, and blunted SIRT1-mediated anti-apoptotic and anti-oxidative effects and cardioprotection.

**Conclusions:**

Overexpression of SIRT1 reduces diabetes-exacerbated MI/R injury and oxidative stress via activating eNOS in diabetic rats. The findings suggest SIRT1 may be a promising novel therapeutic target for diabetic cardiac complications.

## Background

Type 2 diabetes mellitus has reached epidemic proportions in most developed and many developing nations [1]. Numerous clinical studies have shown that cardiovascular diseases are the leading cause of morbidity and mortality among diabetic patients [2]. People with diabetes have two to five times higher risk of developing ischemic heart disease than nondiabetic population [3]. Diabetic patients are more susceptible to myocardial ischemic injury than non-diabetic patients, with worse clinical outcomes and greater mortality [4–6]. In recent years, the pursuit for novel rescue approaches that are effective in diabetic ischemic myocardium has significantly increased. In particular, identifying the molecular basis linking diabetes with increased susceptibility to ischemic injury is not only scientifically important, but may reveal potential new therapeutic targets against ischemic heart diseases under diabetic condition.

Silent information regulator 1 (SIRT1) is a nicotinamide adenine dinucleotide (NAD^+^)-dependent histone deacetylase involved in the regulation of metabolism, cell survival, differentiation, and longevity [7, 8]. SIRT1 exerts beneficial effects on glucose-lipid homeostasis and insulin sensitivity in diabetes from both animal studies and clinical research [9, 10]. Importantly, endogenous SIRT1 is involved in cardioprotection [11]. SIRT1 expression was decreased in ischemic/reperfused hearts compared with sham hearts. Overexpression of SIRT1 reduced myocardial ischemia/reperfusion (MI/R) injury in the heart, while cardiac specific downregulation of SIRT1 promoted myocardial injury following MI/R [12]. Both gain- and loss-of-function experiments suggest that SIRT1 makes the heart more resistant to MI/R injury.

Recently, several studies have indicated that SIRT1 expression was significantly reduced in diabetic heart [13, 14], we therefore hypothesized that up-regulation of cardiac SIRT1 in the diabetic condition may overcome the increased susceptibility of the diabetic heart to I/R injury. To test this hypothesis, we employed adenoviral vectors to selectively up-regulate the expression of SIRT1 in high-fat diet-fed and streptozotocin-induced (HFD-STZ) type 2 diabetic heart, then investigate whether this intervention was sufficient to reduce MI/R injury in diabetic rats and further explore the underlying mechanisms.

## Methods

### Induction of type 2 diabetic model

All animal experiments were conducted in accordance with the National Institutes of Health Guidelines for the Care and Use of Laboratory Animals (NIH Publication No. 85–23, revised, 1996). All study protocols were approved by the Fourth Military Medical University Committee (Shaanxi, China). The high-fat diet-fed and streptozotocin-induced (HFD-STZ) type 2 diabetic rat model was developed as described in our previous studies [15, 16] by providing HFD (D12451, Research Diets, NJ, USA) containing 45 % fat (kcal %), 35 % carbohydrate, and 20 % protein for 4 weeks and then one-shot injection of STZ (40 mg/kg i.p.) (Sigma, St. Louis, MO, USA). HFD was continuously fed after STZ injection, and then hyperglycemic rats (fasting blood glucose ≥11.1 mmol/L, from at least three samplings) 1 week after STZ injection were considered to have developed type 2 diabetes and were studied. The mortality rate of rats after STZ administration was 11.1 % (8 of 72 total rats died). A normal non-diabetic control group was included and fed with a control diet (10 % fat, 20 % protein, and 70 % carbohydrates, D12450H, Research Diets).

### Cardiac overexpression of SIRT1 by adenovirus infection in vivo

Replication-deficient recombinant adenoviral vectors encoding SIRT1 were generated using the Ad-Easy system. In brief, the cDNA for SIRT1 was cloned into pMD19-T simple vector and then transferred into pAdTrack-CMV, resulting in pAdTrack-SIRT1. The shuttle vectors were used to generate recombinant adenoviral vectors encoding SIRT1 (Ad-SIRT1). Adenoviral vectors encoding green fluorescent protein (Ad-GFP) were used as control. Adenoviruses were purified by centrifugation and the adenoviral titer was determined by plaque assays.

**Fig. 1:**
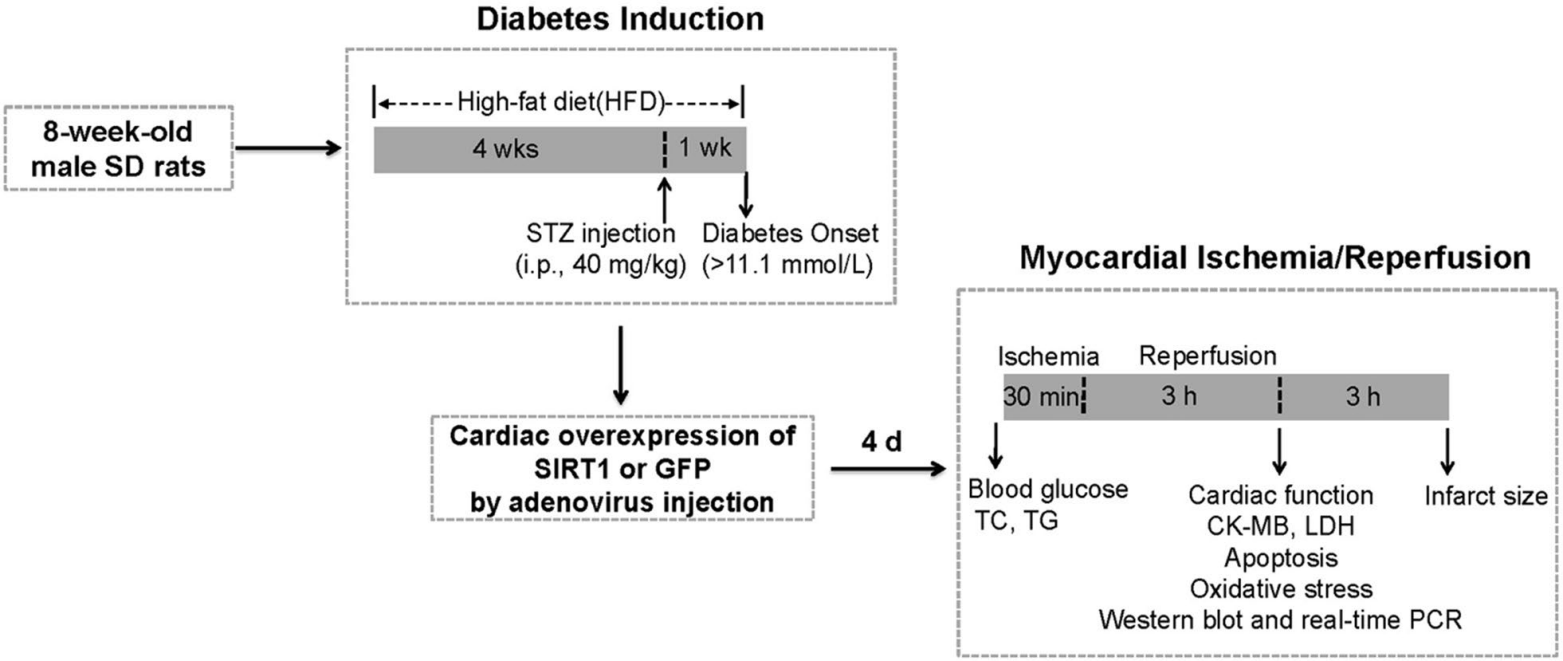
Schematic representation of experimental protocol. Diagram illustrates the diabetic induction period, adenoviral injection time point, ischemia/reperfusion period, and data acquisition time points. *STZ* streptozotocin, *TC* total cholesterol, *TG* triacylglycerol, *CK-MB* creatine kinase–MB, *LDH* lactate dehydrogenase

The adenoviral-mediated gene delivery was performed as previously described [17, 18]. The rats were anesthetized with 3 % pentobarbital sodium (60 mg/kg), and the heart was exposed through a left thoracotomy at the fourth rib. Adenoviral vectors (3 × 10^10^ pfu per rat) were administered by direct injection into the left ventricular free wall with a 30-gauge needle. The rats received intramyocardial injections of adenovirus expressing SIRT1 or GFP at five sites separated by about 2–3 mm around the left anterior descending (LAD) coronary artery. Four days after gene transfer, fasting blood samples were collected to analyze blood glucose, serum TC (total cholesterol) and TG (triacylglycerol) levels before the rats were subjected to MI/R (Fig. 1). The levels of serum TC and TG were analyzed using an automatic biochemical analyzer (Advia 2400, Siemens, Germany).

### Myocardial ischemia–reperfusion model

On the fourth day after adenoviral injection, the rats were re-anesthetized by intraperitoneal (i.p.) administration of 3 % pentobarbital sodium. Myocardial ischemia was induced by re-opening the chest followed by a slipknot (6–0 silk suture) around the LAD coronary artery about 2–3 mm near its origin. Regional myocardial ischemia was verified by the development of a pale color in the ischemic area and changes of electrocardiogram (ST-segment elevation). The slipknot was loosened after 30 min of ischemia, and the ischemic myocardium was reperfused for 3 h. In sham rats, the silk suture was passed underneath the LAD artery without ligation.

Age-matched normal and diabetic male rats were randomly assigned into five experimental groups: (1) NS—non-diabetic sham rats; (2) NIR—non-diabetic rats receiving Ad-GFP were subjected to I/R; (3) DS—diabetic sham rats; (4) DIR—diabetic rats receiving Ad-GFP were subjected to I/R; (5) Ad.SIRT1—diabetic rats receiving Ad-SIRT1 were subjected to I/R. There are eight animals in each group.

### Assessment of SIRT-1 activity

SIRT-1 activity in myocardial tissue was measured by a fluorometric assay kit (Cyclex, Japan). The principle of the kit is to determinate protease activity after modulating the protein through SIRT-1-mediated deacetylation. The peptide is labeled with fluorophore and quencher. The activity of SIRT-1 is directly proportional to the amount of fluorescence emitted by proteolytic cleavage of deacetylated peptide. Data were expressed as percentage activity.

### Cardiac functional assessment

Cardiac function was monitored continuously during the whole period of I/R. A microcatheter was inserted into the left ventricular cavity under anesthesia through the right common carotid artery to assess the left ventricular developed pressure (LVDP). Hemodynamic data were recorded on a polygraph (RM-6240C; Chengdu Instrument Co,. LTD, China). The maximal rate of rise and decline of left ventricular pressure (±LV dP/dt_max_) were derived by digital computer algorithms.

### Quantification of myocardial infarct size

After 6 h of reperfusion, myocardial infarct size (INF) was assessed as described before [16, 19]. LAD coronary artery was retied and 1.5 ml of 2 % Evans blue dye was perfused into the LV cavity to stain the non-ischemic region (area not at risk). The heart was rapidly excised and frozen at −20 °C. The frozen heart was cut into 1–2 mm thick sections perpendicular to the long axis of the heart. Slices were stained with 1 % triphenyltetrazolium chloride (TTC) in phosphate buffer (pH 7.4) for 15 min at 37 °C. TTC-unstained pale area (infarct zone), TTC-stained red area (ischemic but viable myocardium) and Evans blue-unstained regions (area-at-risk, AAR) were analyzed by using an image analysis system (Image Pro Plus 6.0; Media Cybernetics). Myocardial infarct size was determinated as a percent of infarct zone (INF) over total AAR (INF/AAR × 100 %).

### Determination of serum creatine kinase-MB and lactate dehydrogenase

After the 3 h reperfusion period, blood samples were collected from the carotid artery. Serum creatine kinase-MB (CK-MB) and lactate dehydrogenase (LDH) levels were determined with the use of commercial kits (Nanjing jiancheng Bioengineering, China). The activities of these two enzymes were expressed as U/L.

### Quantification of cardiomyocyte apoptosis

Cardiomyocyte apoptosis in the area at risk (AAR) has a significant effect on myocardial survival and function. After 3 h of reperfusion, tissue samples from the area at risk (AAR) were analyzed. Cardiomyocyte apoptosis was detected by terminal deoxynucleotidyl nick-end labeling (TUNEL) and caspase-3 activity assay. TUNEL labeling was performed as described in previous study [6, 20] by using an in situ cell death detection kit (Roche). In brief, the slides were incubated with TUNEL reaction mixture and then counterstained with the 4′,6-diamino-2-phenylindole (DAPI) to detect the nuclei. The apoptosis index was calculated as a percentage of the number of TUNEL-positive apoptotic cells over the total number of nucleated cells (DAPI staining). Myocardial caspase-3 activity was determined as described before [21] by using a caspase colorimetric assay kit (Chemicon, Temecula, CA, USA) according to manufacturer’s protocol.

### Quantification of superoxide generation, malonaldialdehyde (MDA) and total superoxide dismutase (SOD)

Lucigenin-enhanced chemiluminescence was used to assess superoxide production in heart tissue as described previously [22]. The results were expressed as relative light units (RLU) per second per milligram tissue weight (RLU/mg/s). The level of MDA and the activities of total SOD in myocardial homogenates were determined spectrophotometrically as previously described [21, 23] with the use of commercial kits (Nanjing jiancheng Bioengineering, China).

### Myocardial eNOS activity

The AAR of rat hearts was homogenized in 0.9 % NaCl (1:10, wt/vol). The tissue homogenate was centrifuged at 12,000*g* for 10 min at 4 °C, and the supernatant was collected to determine myocardial eNOS activity using a spectrophotometrical assay kit (Nanjing Jiancheng Bioengineering) as previously reported [21, 24].

### Western blotting

Frozen AAR samples were homogenized in lysis buffer and protein concentrations were determined by using a BCA protein assay. After separated by electrophoresis on sodium dodecyl sulfate polyacrylamide gel electrophoresis (SDS-PAGE), the proteins were transferred to nitrocellulose membranes. The membranes were blocked with 5 % skim milk for 1 h at room temperature and then incubated with the appropriate primary antibodies including anti-SIRT1, anti-eNOS phosphorylation (p-eNOS) at Ser 1177, anti-eNOS, anti-gp91^phox^ (Cell Signaling Technology) and anti-GAPDH (Wuhan Boster Biological Technology Ltd.) overnight at 4 °C. After three washing with phosphate buffer solution with tween-20 (PBST), the membrances were incubated with corresponding horseradish peroxidase-conjugated second antibodies. After additional PBST washes, the blots were evaluated by using an enhanced chemiluminescent system.

### Quantitative real-time PCR

Total RNA was extracted with Trizol reagent (Invitrogen, Shanghai, China) and was subjected to reverse transcription using superscript^®^ III first-strand systhesis system (Invitrogen, Shanghai, China) following the manufacturer’s instruction. Real-time PCR was carried out using Power SYBR green PCR master mix (Bio-rad). The following primer sequences were used: SIRT1 forward GCAGGTTGCAGGAATCCAAA, reverse GGCAAGATGCTGTTGCAAAG; GAPDH forward GACATGCCGCCTGGAGAAAC, reverse AGCCCAGGATGCCCTTTAGT. Data were normalized relative to those for GAPDH expression using the 2^−ΔΔCt^ method.

### eNOS acetylation expression

Acetylated eNOS antibody was not commercially available up to now. The expression of eNOS acetylation was determined by co-immunoprecipitation assay as described in our previous study [24]. eNOS (1:1000 crosslinked to magnetic beads (Dynal, Invitrogen) for extraction) was immunoprecipitated from 40 μg of myocardial tissue lysate, and the primary antibody for acetyl-lysine (Cell signaling) was employed to detect the association of the acetyl-lysine with eNOS by using immunoblotting.

### Antagonize NOS with N-nitro-l-arginine methyl ester (L-NAME)

After adenoviral injection, eight rats from the Ad.SIRT1 group were given L-NAME in their drinking water (200 mg/l) for 4 days before being subjected to MI/R (Ad.SIRT1 + L-NAME group). The average dosage of L-NAME was nearly 15 mg/kg per day. This dose of L-NAME was based on the early studies [25, 26] in which near-maximal inhibition of NOS in the body was achieved.

### Statistical analysis

All values are presented as mean ± SEM. Differences among comparisons were evaluated with one-way ANOVA followed by Bonferroni corrected t test where appropriate. P-values less than 0.05 were taken as statistically significant. Statistical tests were performed using GraphPad Prism software version 5.0 (GraphPad Software, Inc., San Diego, CA, USA).

## Results

### Characterization of animals

As shown in Table 1, compared with the non-diabetic rats, diabetic animals manifested significantly increased blood glucose, serum TC and TG, and decreased body weight, indicating that type 2 diabetic model was created in this study. Delivery of Ad-GFP or Ad-SIRT1 into the diabetic rat hearts did not show any significant effect on these parameters.

**Table 1:** Characterization of animals

Groups	Non-DM	DM	DM + Ad-GFP	DM + Ad-SIRT1
Glucose (mmol/L)	4.9 ± 0.6	18.6 ± 2.6*	20.1 ± 2.9*	19.1 ± 3.1*
TC (mmol/L)	1.42 ± 0.13	4.74 ± 0.16**	4.89 ± 0.21**	4.71 ± 0.24**
TG (mmol/L)	0.59 ± 0.08	1.21 ± 0.09*	1.36 ± 0.10*	1.41 ± 0.11*
Body weight (g)	281 ± 19	230 ± 17*	224 ± 18*	221 ± 16*

Values presented are mean ± SEM. n = 9

*DM* high-fat diet-fed streptozotocin (HFD-STZ) diabetic group, *TC* total cholesterol, *TG* triacylglycerol

* *P* < 0.05, ** *P* < 0.01 vs. Non-DM

### SIRT1 expression and activity were decreased in diabetic hearts

**Fig. 2:**
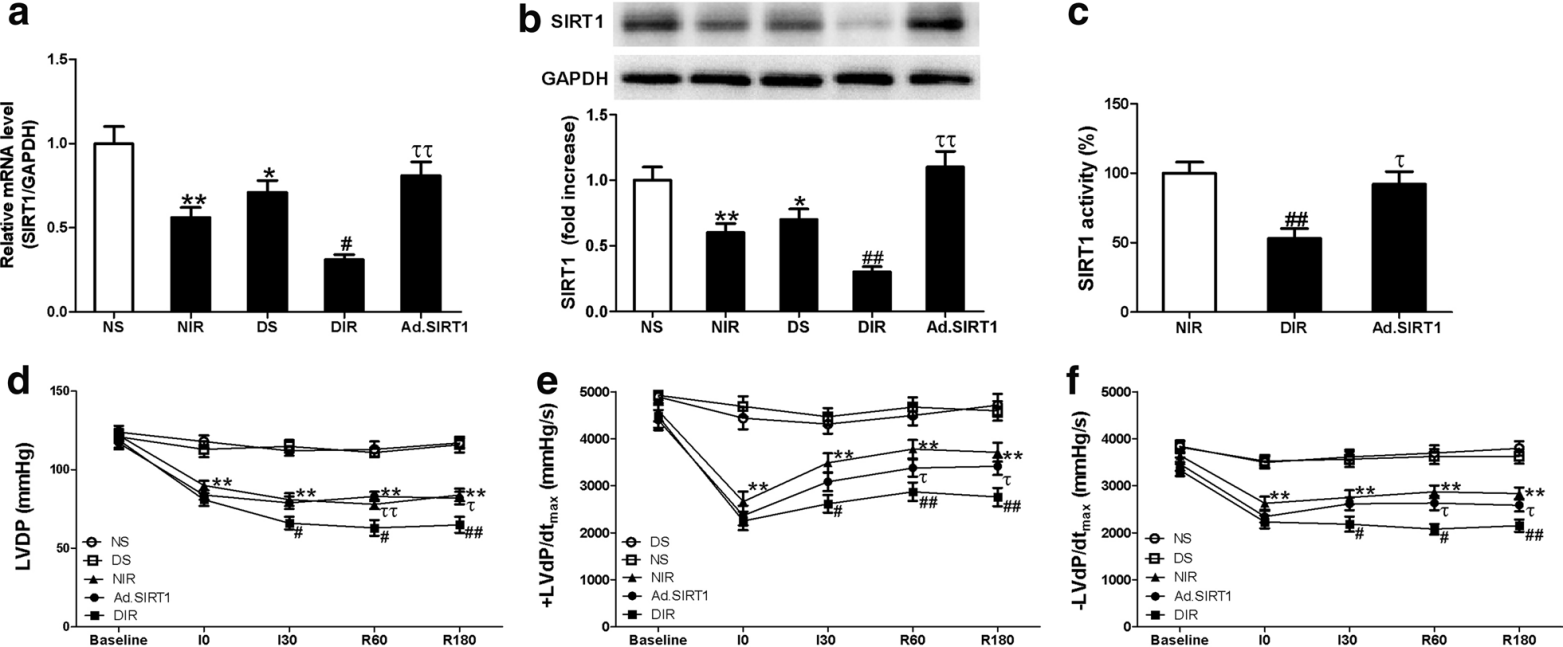
Up-regulation of SIRT1 improved cardiac function after MI/R in diabetic rats. **a** mRNA expression of SIRT1. **b** Protein expression of SIRT1. **c** SIRT1 activity. **d** LVDP, left ventricular developed pressure. **e**, **f** ±LV dP/dtmax, the instantaneous first derivation of left ventricle pressure. NS, non-diabetic sham rats; NIR, non-diabetic I/R rats receiving Ad-GFP; DS diabetic sham rats; DIR diabetic I/R rats receiving Ad-GFP; Ad.SIRT1, diabetic I/R rats receiving Ad-SIRT1. Values are mean ± SEM. n = 8. *P < 0.05, **P < 0.01 vs. NS; ^#^ *P* < 0.05, ^##^ *P* < 0.01 vs. NIR; ^τ^ *P* < 0.05, ^ττ^ *P* < 0.01 vs. DIR

mRNA and protein expression of myocardial SIRT1 were significantly reduced in diabetic rats compared to control non-diabetic animals (DS vs. NS or DIR vs. NIR, *P* < 0.05 or *P* < 0.01, Fig. 2a, b). Myocardial SIRT1 expression was also decreased after MI/R in control and diabetic rats compared with sham animals respectively (*P* < 0.05 or *P* < 0.01). Moreover, myocardial SIRT-1 activity was decreased in diabetic MI/R rats (DIR) compared to non-diabetic MI/R (NIR) animals. These results suggested that diabetes or I/R alone could reduce SIRT1 expression, and significant down-regulated SIRT1 expression was induced in diabetic I/R hearts.

### Up-regulation of SIRT1 improved cardiac function in diabetic MI/R animals

As shown in Fig. 2, there were no significant differences in LVDP and ±LV dP/dt_max_ between non-diabetic sham (NS) and DM sham (DS) rats during I/R period. However, after MI/R, diabetic rats (DIR) showed aggravated myocardial functional impairment compared with non-diabetic animals (NIR) as evidenced by decreased LVDP and ±LV dP/dt_max_ in the course of I/R (n = 8, *P* < 0.05 or *P* < 0.01). Delivery of Ad-SIRT1 markedly increased SIRT1 expression and activity in diabetic MI/R hearts (Fig. 2a–c). Compared with Ad-GFP-treated diabetic MI/R rats (DIR), up-regulation of SIRT1 (Ad-SIRT1) significantly elicited a significant recovery in LVDP and ±LV dP/dt_max_ in diabetic MI/R rats (n = 8, *P* < 0.05 or *P* < 0.01, Fig. 2c–e). No significant difference was found in the changes of heart rate (HR) among all the groups (data not shown). These results showed that up-regulation of SIRT1 alleviated cardiac dysfunction in diabetic rats subjected to MI/R.

### Up-regulation of SIRT1 attenuated MI/R injury in diabetic rats

**Fig. 3:**
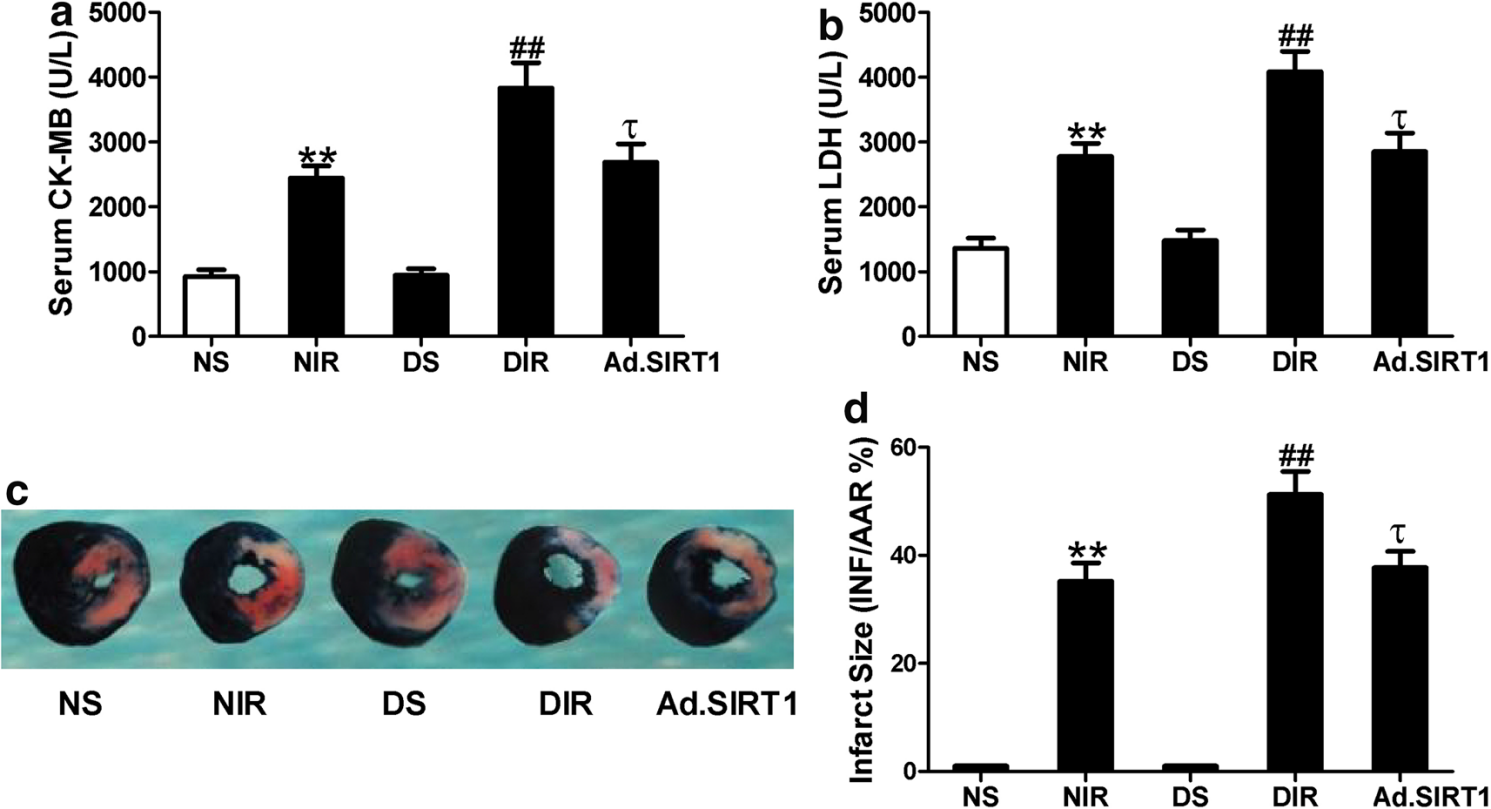
Up-regulation of SIRT1 reduced MI/R injury in diabetic rats. **a** Serum creatine kinase-MB (CK-MB) levels. **b** Serum lactate dehydrogenase (LDH) levels. **c** Representative photos of myocardial infarct size. **d** Myocardial infarct size expressed as a percent of infarct area (INF) over total area at risk (AAR). NS, non-diabetic sham rats; NIR, non-diabetic I/R rats receiving Ad-GFP; DS, diabetic sham rats; DIR, diabetic I/R rats receiving Ad-GFP; Ad.SIRT1, diabetic I/R rats receiving Ad-SIRT1. Values are mean ± SEM. n = 8. **P < 0.01 vs. NS; ^##^ *P* < 0.01 vs. NIR; ^τ^ *P* < 0.05 vs. DIR

To determine whether up-regulation of SIRT1 might reduce myocardial injury, serum CK-MB and LDH levels and myocardial infarct size were measured. Compared with non-diabetic sham (NS) animals, there were obvious myocardial infarction and increased serum CK-MB and LDH levels in non-diabetic MI/R (NIR) rats, which indicated injury on cell membrane of cardiomyocytes after I/R leading to the release of cell content. The effects of Ad.SIRT1 on non-diabetic MI/R injury were assessed in our preliminary study. It was found that overexpression of SIRT1 significantly reduced infarct size and serum CK-MB levels in non-diabetic rats (infarct size: 22.8 ± 2.9 vs. 35.2 ± 3.4 in NIR group, CK-MB: 1687 ± 154 vs. 2438 ± 189 in NIR group, n = 8, *P* < 0.05 or *P* < 0.01). As shown in Fig. 3, larger infarct size and further increased serum CK-MB and LDH levels were observed in diabetic MI/R (DIR) rats (n = 8, *P* < 0.05), suggesting that diabetes aggravated MI/R injury. Up-regulation of SIRT1 significantly reduced infarct size and serum CK-MB and LDH levels in diabetic rats (Infarct size: 37.7 ± 3.1 vs. 51.3 ± 4.2 in DIR group, n = 8, *P* < 0.05) to the extent as in non-diabetic animals following MI/R (Infarct size: 37.7 ± 3.1 vs. 35.2 ± 3.4 in NIR group, n = 8, *P* > 0.1).

**Fig. 4:**
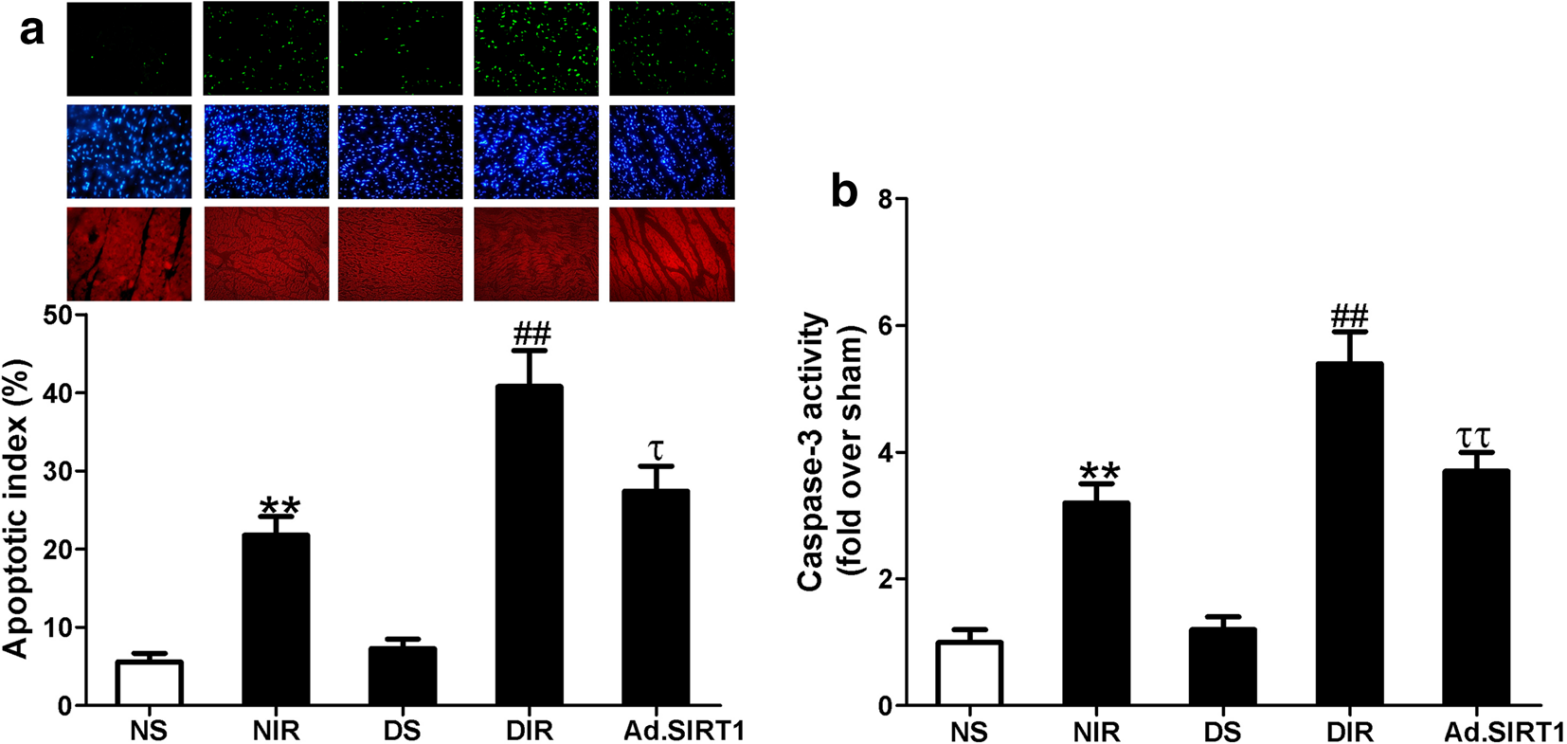
Up-regulation of SIRT1 inhibited cardiomyocyte apoptosis in diabetic hearts subjected to I/R. **a** *Top* Representative photomicrographs of TUNEL-positive apoptotic cardiomyocytes in the area at risk following MI/R (original magnification ×400). *Bottom* Quantitative analysis of myocardial apoptosis. **b** Myocardial caspase-3 activity. NS, non-diabetic sham rats; NIR, non-diabetic I/R rats receiving Ad-GFP; DS, diabetic sham rats; DIR, diabetic I/R rats receiving Ad-GFP; Ad.SIRT1, diabetic I/R rats receiving Ad-SIRT1. Values are mean ± SEM. n = 8. **P < 0.01 vs. NS; ^##^ *P* < 0.01 vs. NIR; ^τ^ *P* < 0.05, ^ττ^ *P* < 0.01 vs. DIR

Apoptosis is another major form of cell death after I/R, we then investigate whether up-regulation of SIRT1 could decrease cardiomyocyte apoptosis induced by MI/R. Compared with non-diabetic sham (NS) animals, the percentage of TUNEL-positive cells (Apoptotic index: 21.8 ± 2.4 % of NIR vs. 5.6 ± 1.1 % of NS, Fig. 4a, n = 8, *P* < 0.01) and myocardial caspase-3 activity were significantly increased in the non-diabetic I/R (NIR) group. Similarly, diabetic MI/R rats showed further increased myocardial apoptotic index and caspase-3 activity compared with NIR group. Up-regulation of SIRT1 significantly reduced myocardial apoptosis in diabetic rats to the extent as in non-diabetic animals following MI/R (Apoptotic index: 27.4 ± 3.2 % of Ad.SIRT1 vs. 40.8 ± 4.6 % of DIR, Fig. 4a, n = 8, *P* < 0.01). All these results proved that up-regulation of SIRT1 reduced myocardial susceptibility to I/R injury in diabetic rats, which contributed to the improvement of cardiac function after MI/R.

### Up-regulation of SIRT1 attenuated I/R induced oxidative stress in diabetic rats

**Fig. 5:**
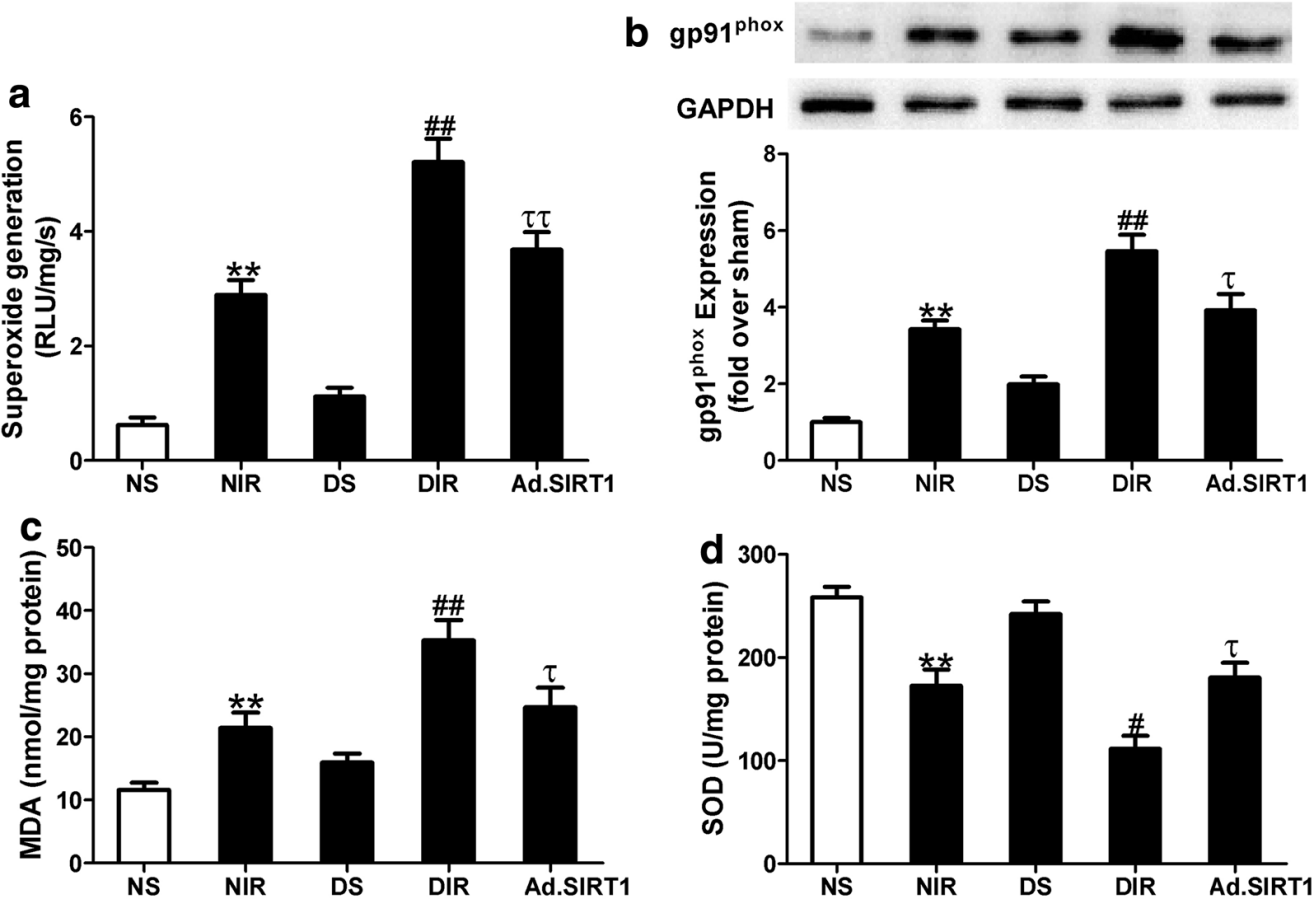
Up-regulation of SIRT1 attenuated oxidative stress in diabetic MI/R rats. **a** Cardiac superoxide generation. **b** *Top* representative blot images. *Bottom* quantitative analysis of gp91^phox^ expression. **c** MDA, malondialdehyde contents. **d** *SOD* superoxide dismutase activity. NS, non-diabetic sham rats; NIR, non-diabetic I/R rats receiving Ad-GFP; DS, diabetic sham rats; DIR, diabetic I/R rats receiving Ad-GFP; Ad.SIRT1, diabetic I/R rats receiving Ad-SIRT1. Values are mean ± SEM. n = 4–6. **P < 0.01 vs. NS; ^#^ *P* < 0.05, ^##^ *P* < 0.01 vs. NIR; ^τ^ *P* < 0.05, ^ττ^ *P* < 0.01 vs. DIR

Compared with non-diabetic MI/R (NIR) group, myocardial superoxide generation was significantly increased in diabetic MI/R (DIR) group (*P* < 0.01), and up-regulation of SIRT1 inhibited superoxide accumulation (Fig. 5a, n = 6, *P* < 0.01 vs. DIR group). gp91^phox^ is a major component of NADPH oxidase, which is the most important superoxide-producing enzyme. Up-regulation of SIRT1 markedly reduced gp91^phox^ expression in diabetic MI/R rats (Fig. 5b, n = 4, *P* < 0.05 vs. DIR group). Moreover, the level of MDA was determined as a biomarker of oxidative stress. There was a marked increase in MDA production in diabetic MI/R (DIR) group in comparison with the non-diabetic MI/R (NIR) group, which was reduced by Ad-SIRT1 administration (Fig. 5c). In contrast, Ad-SIRT1 administration increased myocardial antioxidant enzyme SOD activity in diabetic MI/R rats (Fig. 5d). These results suggested that SIRT1 reduced I/R-stimulated oxidative stress in diabetic rats.

### Up-regulation of SIRT1 protects the diabetic heart against I/R injury via the modulation of eNOS activity

**Fig. 6:**
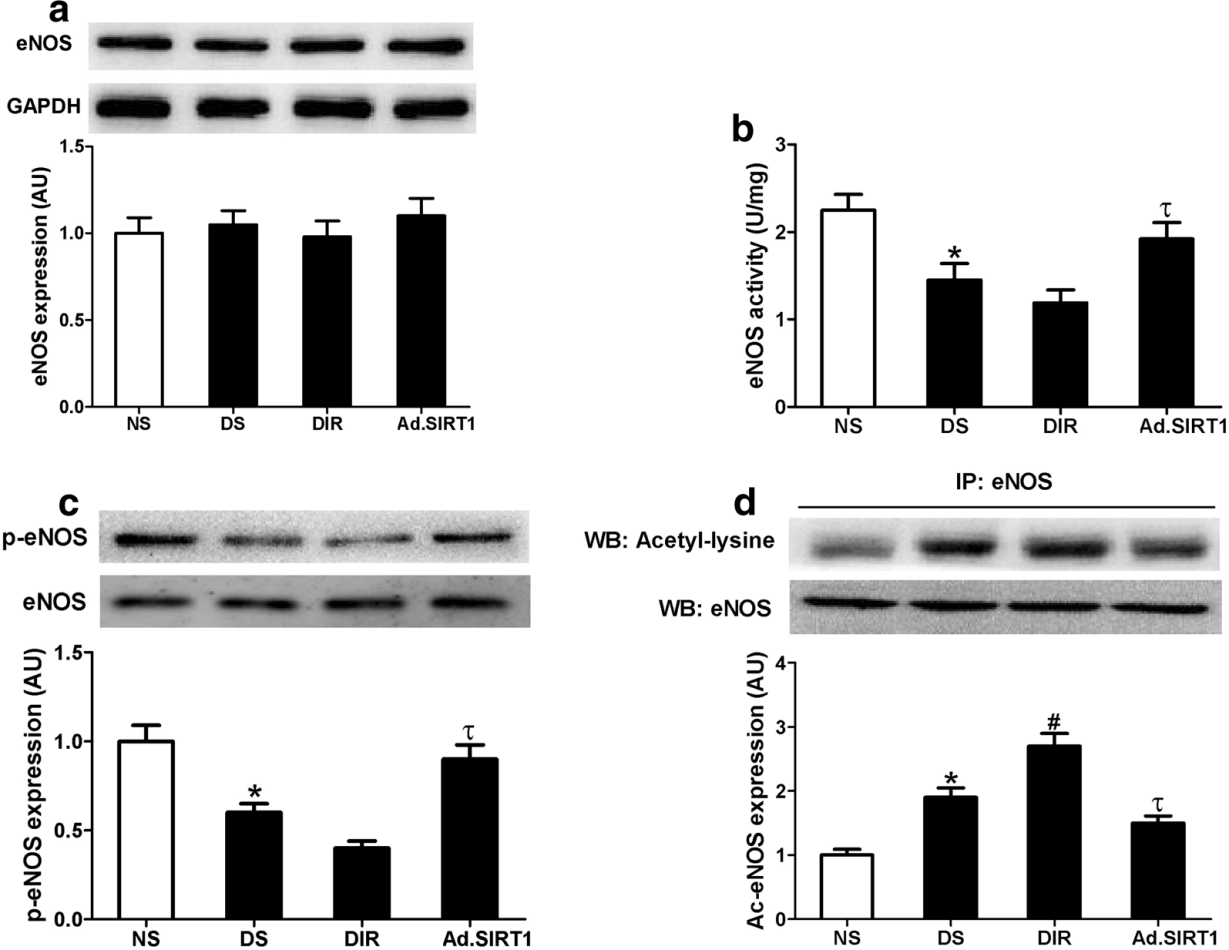
SIRT1 overexpression increased eNOS activity and eNOS phosphorylation, and attenuated eNOS acetylation in diabetic heart. **a** *Top* representative blot images. *Bottom* quantitative analysis of eNOS expression; **b** eNOS activity. **c**, **d** *Top* representative blot images. *Bottom* quantitative analysis of phosphorylated eNOS (p-eNOS) and acetylated eNOS (Ac-eNOS). NS, non-diabetic sham rats; DS, diabetic sham rats; DIR, diabetic I/R receiving Ad-GFP rats; Ad.SIRT1, diabetic I/R receiving Ad-SIRT1 rats. Values are mean ± SEM. n = 4–6. *P < 0.05 vs. NS; ^#^ *P* < 0.05 vs. DS; ^τ^ *P* < 0.05 vs. DIR

It has been proven that activation of eNOS exerts beneficial effects on I/R hearts [27, 28]. To further investigate the mechanisms underlying the SIRT1-mediated cardioprotection, we therefore measured eNOS activity and phosphorylated eNOS at Serine 1177 (activation state) and acetylated eNOS (deactivation state) in the hearts. As shown in Fig. 6, there was no significant difference in eNOS expression among all the groups, while eNOS activity was significantly decreased in diabetic heart (DS) compared with that in non-diabetic control (NS). Western blot analysis demonstrated decreased eNOS phosphorylation and increased eNOS acetylation in diabetic heart. Ad-SIRT1 administration significantly increased eNOS activity and eNOS phosphorylation, and attenuated eNOS acetylation in diabetic heart (n = 4–6, *P* < 0.05 vs. DIR group). These results suggested that up-regulation of SIRT1 might increase eNOS activity by enhanced phosphorylation of eNOS and reduced acetylation of eNOS.

**Fig. 7:**
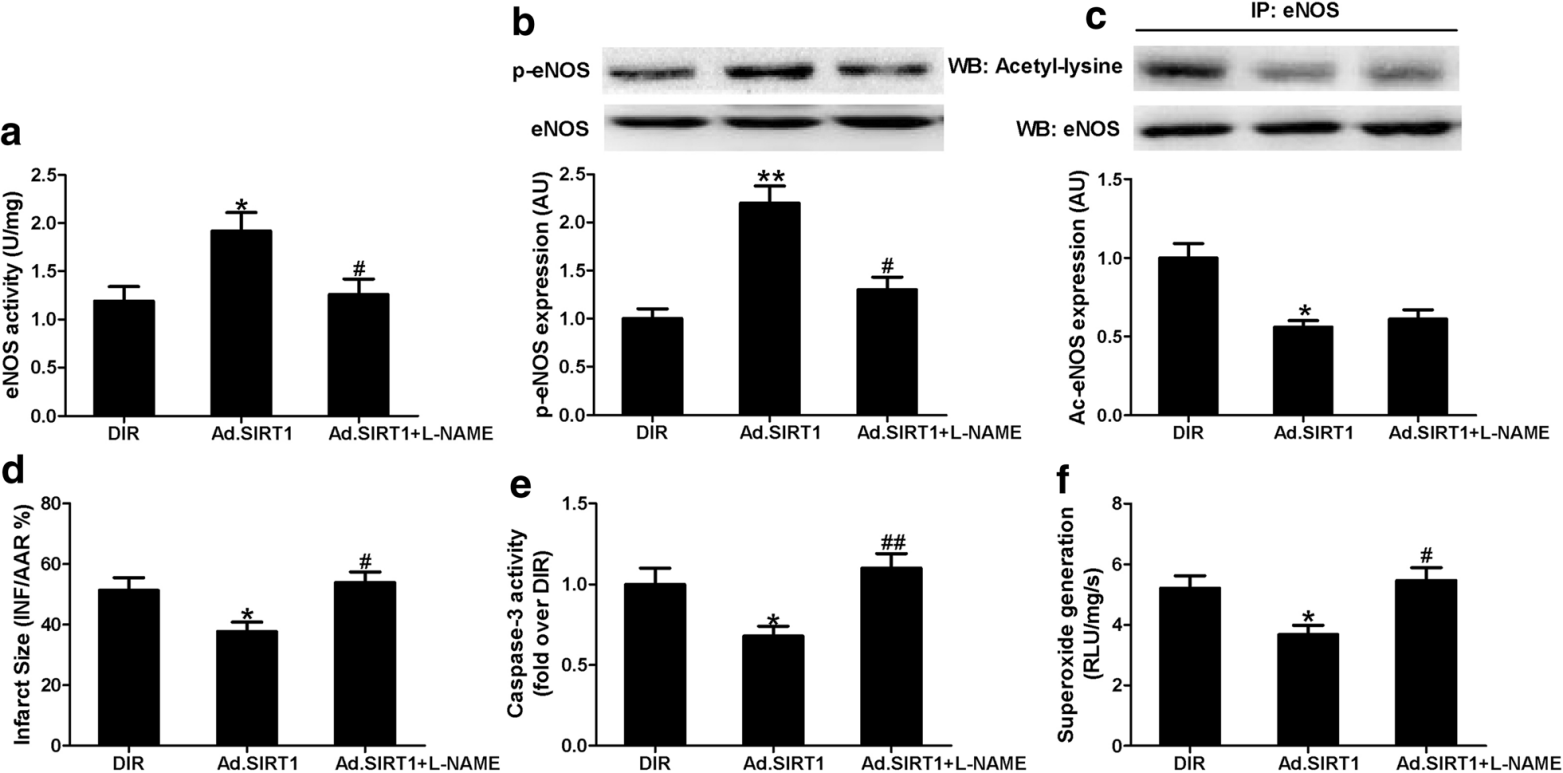
NOS inhibitor L-NAME inhibited cardioprotective effects of Ad-SIRT1. **a** eNOS activity, **b** *Top* representative blot images. *Bottom* quantitative analysis of phosphorylated eNOS (p-eNOS); **c** *Top* representative blot images. *Bottom* quantitative analysis of acetylated eNOS (Ac-eNOS); **d** Myocardial infarct size expressed as a percent of infarct area (INF) over total area at risk (AAR); **e** Myocardial caspase-3 activity; **f** Cardiac superoxide generation. DIR, diabetic I/R rats receiving Ad-GFP; Ad.SIRT1, diabetic I/R rats receiving Ad-SIRT1; Ad.SIRT1 + L-NAME, diabetic I/R rats receiving Ad-SIRT1 and L-NAME. Values are mean ± SEM. n = 4–6. *P < 0.05, **P < 0.01 vs. DIR; ^#^ *P* < 0.05, ^##^ *P* < 0.01 vs. Ad.SIRT1

To further ascertain the degree of involvement of eNOS in the cardioprotective effects of Ad-SIRT1, NOS inhibitor L-NAME was administered after adenoviral injection. As shown in Fig. 7, co-treatment with L-NAME significantly blocked eNOS phosphorylation and reduced eNOS activity although did not change acetylation of eNOS. Moreover, the cardioprotective effects of Ad-SIRT1, as previous evidence by decreased myocardial infarct size and caspase-3 activity and superoxide generation in diabetic rats, were abolished by L-NAME (Fig. 7d–f). These results suggested that up-regulation of SIRT1 attenuates MI/R-induced myocardial injury possibly via modulating eNOS activity.

## Discussion

In this study, we have demonstrated that SIRT1 is a powerful regulator in diabetic MI/R injury. This conclusion is based on several novel findings. First, we have provided evidences that SIRT1 expression was decreased in diabetic heart, and overexpression of SIRT1 alleviated MI/R injury and improved cardiac function in diabetic rats. Second, SIRT1-mediated cardioprotection involved inhibition of oxidative stress. Third, the mechanisms of the cardioprotection were mediated by modulation of eNOS activity.

Overwhelming epidemiological and clinical data have demonstrated that the diabetic heart is more sensitive to I/R injury [6, 29, 30]. It has been demonstrated that diabetes mellitus can exacerbate MIR injury and blunt the protective effect of various therapeutic agents [31, 32]. Thus, novel strategies and targets are urgently needed to reduce myocardial susceptibility to I/R injury in diabetic state. To address this issue, high-fat diet-fed and streptozotocin-induced (HFD-STZ) type 2 diabetic animal model was developed in the present study. HFD elicited insulin resistance and STZ administration reduced insulin levels, such that the animals were unable to maintain normal glucose levels and develop hyperglycemia. This model has been demonstrated to be suitable for studying the pathophysiology of type 2 diabetes as well as for testing agents for the treatment of type 2 diabetes in several studies [16, 33, 34]. Markedly increased blood glucose level was also found in our diabetic animals. After developing this type 2 diabetic animal model, we observed that the diabetic rats showed aggravated MI/R injury and more severe myocardial functional impairment compared with non-diabetic animals, which was consistent with the previous study [31].

Diabetes mellitus disrupts the cardiac gene expression profile, which might be involved in the development of cardiac implications [35]. SIRT1 is proved to be an endogenous protective molecule against MI/R-induced injury [12]. Therefore, we focused on the alteration of SIRT1 expression in diabetic heart. Our study indicated that mRNA and protein expression of myocardial SIRT1 were significantly decreased in HFD-STZ-induced type 2 diabetic rats. Previous studies have demonstrated that SIRT1 expression was reduced in STZ-induced type 1 diabetic animals [14, 36]. Based on these data, it’s speculated that SIRT1 might be partly reduced by hyperglycemia, which is a common characteristic between type 1 and type 2 diabetes.

It has been reported that resveratrol has the ability to stimulate SIRT1 and protects against diabetic cardiomyopathy in experimental animals [14, 36]. However, resveratrol is not a direct activator of SIRT1 [37]. Some studies showed that resveratrol exerted its cardioprotective effects through multiple mechanisms including activation of AMPK pathway and modulation of calcium handling proteins [38, 39]. Moreover, resveratrol was found to improve whole-body glucose metabolism and decrease blood glucose level in diabetic animals [14], which may account for the cardioprotective effects of resveratrol. Our study indicated that cardiac overexpression of SIRT1 by adenovirus infection did not have significant influence on blood glucose and serum TC and TG. Importantly, overexpression of myocardial SIRT1 alleviated cardiac dysfunction and myocardial injury (as evidenced by increased ±LV dP/dt_max_ and decreased serum CK-MB/LDH activities, myocardial infarction and cardiomyocyte apoptosis) in diabetic rats to the extent as in non-diabetic animals subjected to MI/R, suggesting that cardiac specific up-regulation of SIRT1 is sufficient to reduce myocardial susceptibility to I/R injury in diabetic rats.

Myocardial oxidative stress contributes importantly to vulnerability of diabetic myocardium to I/R injury [6, 40]. As expected, diabetes enhances oxidative stress and reduces antioxidant defenses in I/R hearts as evidenced by increased superoxide generation and reduced SOD activity. Overexpression of SIRT1 not only decreased the generation of superoxide content but also increased antioxidant enzyme SOD activity in the diabetic I/R heart tissue. NADPH oxidase is an important source of superoxide anion production [41]. Ad-SIRT1 administration decreased myocardial gp91^phox^ (a critical component of NADPH oxidase) expression in diabetic I/R rats. MDA content in tissue usually reflects the level of oxidative stress [42]. Ad-SIRT1 administration also reduced MDA formation in diabetic I/R heart tissue. Previous studies have reported that SIRT1 increased the expression of MnSOD (mitochondria-specific isoform of SOD) and attenuated mitochondrial oxidative damage induced by myocardial ischemia reperfusion injury [12, 43]. These results suggested that Ad-SIRT1 administration may attenuate both mitochondria and NADPH oxidase-derived Reactive oxygen species (ROS) production in diabetic I/R hearts.

Activation of eNOS has been demonstrated to clearly reduce myocardial infarct size and improve cardiac function in a variety of I/R models [27, 28]. Normally, eNOS phosphorylation and subsequent NO production exert anti-apoptotic and anti-oxidative effects in I/R hearts [44, 45]. Decreased myocardial eNOS–NO availability has been found in both patients and animals with diabetes, which contributes to the development of diabetic complications [46, 47]. Consistent with previous studies, we found reduced eNOS activity in diabetic hearts., while the expression of eNOS was not altered. These results suggest that posttranslational modifications of eNOS may account for the decreased eNOS activity in the setting of diabetes.

SIRT1 is a histone deacetylase that plays an important role in modulating eNOS activity. SIRT1 colocalizes with eNOS, and deacetylation by SIRT1 enhances eNOS activity. Inhibition of SIRT-1 activity can acetylate eNOS and inhibit eNOS activity [48, 49]. In the present study, reduced SIRT1 expression is associated with increased eNOS acetylation (deactivation state) and decreased eNOS phosphorylation (activation state) in diabetic hearts when compared with non-diabetic controls. Moreover, up-regulation of SIRT1 reduced eNOS acetylation and enhanced the phosphorylation of eNOS and the activity of eNOS. These novel findings indicate that SIRT1 is a powerful regulator for maintaining normal eNOS function and active form in diabetic hearts.

To further clarify the role of eNOS in SIRT1-induced cardioprotective effects, NOS inhibitor L-NAME was applied. Co-treatment with L-NAME not only blocked SIRT1-induced eNOS activation, but also attenuated SIRT1-mediated anti-apoptotic and anti-oxidative effects in diabetic MI/R rats, as evidenced by increased myocardial caspase-3 activity and superoxide generation. Moreover, inhibition of eNOS activity blunted the beneficial effects of SIRT1 on myocardial infarct size. These data indicate that SIRT1 may protect the diabetic rat heart against I/R injury via an eNOS-dependent mechanism. It should be noted that SIRT1 participates in cardioprotection via a complex signaling network, including the PGC-1α, AMPK, eNOS and so on [11], and that activation of eNOS observed in our present study may be a dependent pathway by which SIRT1 exerts its cardioprotective effects. Moreover, further studies are required to explore SIRT1 and eNOS effective strength in vitro.

## Conclusions

The current study demonstrated that SIRT1 is a critical regulator in myocardial susceptibility to I/R injury under diabetic condition. Overexpression of SIRT1 reduces diabetes-exacerbated MI/R injury and oxidative stress via modulating eNOS acitivity in diabetic rats. The findings suggest SIRT1 may be a promising novel therapeutic target for diabetic cardiac complications.

## Abbreviations

AAR: area-at-risk
Ad: adenovirus
CK: creatine kinase
GFP: green fluorescent protein
HFD: high-fat diet
INF: myocardial infarct size
LAD: left anterior descending
LDH: lactate dehydrogenase
L-NAME: *N*-nitro-l-arginine methyl ester
±LV dP/dt_max_: maximal rate of rise and decline of left ventricular pressure
LVDP: left ventricular developed pressure
MDA: malonaldialdehyde
MI/R: myocardial ischemia and reperfusion
PBST: phosphate buffer solution with Tween-20
SIRT1: silent information regulator 1
SOD: superoxide dismutase
STZ: streptozotocin
TC: total cholesterol
TG: triacylglycerol
TUNEL: terminal deoxynucleotidyl nick-end labeling
